# Durability of Polymer Metal Multilayer: Focus on the Adhesive Chemical Degradation

**DOI:** 10.3389/fchem.2018.00459

**Published:** 2018-11-22

**Authors:** Florence Dubelley, Corine Bas, Emilie Planes, Emmanuelle Pons, Bernard Yrieix, Lionel Flandin

**Affiliations:** ^1^Univ. Grenoble Alpes, Univ. Savoie Mont Blanc, CNRS, Grenoble INP, LEPMI, Grenoble, France; ^2^EDF R&D, Materials and Mechanics of Components, Moret-sur-Loing, France

**Keywords:** polyurethane, FTIR spectroscopy, hydrolysis, kinetics, delamination

## Abstract

Mechanical toughness and high barriers to air and water may be combined in a polymer–metal multilayer film, provided that the two materials are properly bonded together. Delamination is indeed the most severe flaw observed in service. This suggests that the polyurethane (PU) adhesive at the polymer–metal interface fails to bear the shear forces, as happens principally if a multilayer system is submitted to elevated temperature and humidity. A Raman microscopy of the multilayer revealed a cohesive delamination, with glue on both the surfaces. A detailed investigation of the kinetic of degradation of the polyester was therefore carried out. IR spectroscopy of the standalone PU film hydrolyzed in a controlled manner furnished a series of aging markers. The reference curve was established for approximately a year in continuous severe aging conditions. This curve could be further used to compare the amount of degradation in real systems in a wide range of conditions and time. Moreover, at the metallized interphase, a complex with a free hydroxyl group was detected. The content of this Al^III^ complex based on terephthalate or carbamate increases with the progress of the ester hydrolysis reactionin the layer.

## Introduction

For many packaging applications, the external envelope should prevent the diffusion of gaseous moieties, be welded on the boundaries, and allow general handling. This set of requirements is not generally achievable by a single layer of polymer. Multilayer systems combining the properties of organic or inorganic components with a laminate adhesive are necessary. The barrier properties required for the envelopes of vacuum insulation panels (VIPs) may be realized by either a laminated metal film or a thin metallization. A typical architecture of such envelopes is a polyester film placed for mechanical strength and aluminum protection, and a polyethylene (PE) layer for weldability. The layers are usually glued together with polyurethane (PU) adhesives. The latter provides high bond strength and some barrier performance (Brunner et al., 2006; Brunner and Simmler, 2008). Numerous studies have already been carried on the optimization of the number of layers, their compositions, layouts, and thicknesses (Simmler and Brunner, 2005; Baetens et al., 2010; Garnier et al., 2011; Miesbauer et al., 2014; Pons et al., 2014). At present, they are many different multilayer laminates on the market that include up to more than ten layers and three adhesive layers.

The adhesion between the layers controls the durability of the envelope and thereby that of the entire structure. The strength and performance of the joints can be deteriorated during aging because of the damage in the adhesive substrate interfacial region and the adhesive layer (Fernando et al., 1996; Kinloch et al., 2000; Datla et al., 2012). The interfacial delamination between assembled films is a well-identified problem for extreme barrier applications. For example, delaminations of the PU adhesive layer at PET-Al/PU/PET-Al interfaces have been observed after only 1.5 years of aging at 65°C/75% RH (Brunner and Simmler, 2008). Other authors even evidenced debonding between PET and the sealing interface after 48 and 96 days of aging at 70°C/90% RH, respectively for PE and PP sealant materials (Garnier et al., 2011; Dubelley et al., 2017a).

Two main hypotheses have been formulated to explain the delamination occurring in thin multilayer systems: shear stresses resulting either from the difference in the hygrothermal expansion between the metal and the polymer (Simmler and Brunner, 2005) or by the shrinkage of the polymeric films (Dubelley et al., 2017a). Besides this purely mechanical assumption, it has also been suggested that the PU adhesive might also be degraded with a chemical hydrolysis weakening the interfaces, especially in moist environments (Simmler and Brunner, 2005; Brunner et al., 2006). Both the mechanisms are plausible and could occur concurrently. Hydrolysis has been identified as one of the main mechanisms affecting the PU adhesive (Buchman et al., 1991; Davies and Evrard, 2007; Boubakri et al., 2009; Petrie, 2011; Gac et al., 2013; Zain et al., 2014; Weiss et al., 2016; de Oliveira et al., 2017; Sousa et al., 2018). However, these previous studies are difficult to compare due to the wide variety of PU adhesive formulations.

The present work concerns the hydrothermal degradation of a PU adhesive largely used in polymer–metal multilayer films for packaging applications. The experimental work first concerned the PU individually submitted to accelerating aging in rough static conditions at 70°C/90% RH for ~1 year. The gradual changes in chemistry were revealed by FTIR spectroscopy to identify the most significant degradation markers and monitor the degradation. Multilayer systems were aged in parallel in the same conditions. The chemical changes of the inner PU layer of the laminate could be identified owing to the standalone PU used as the benchmark. The failure mode—adhesive or cohesive—was also identified.

## Materials and methods

### Materials

#### Samples

##### Adhesives

**Scheme 1:**
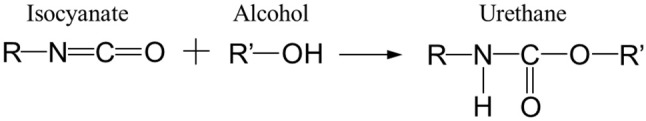
Isocyanate reaction with alcohol.

The adhesive used in the multilayer was a bicomponent PU system obtained by the polyaddition of polyols and polyisocyanates in accordance with the reaction shown in Scheme 1:

In this study, the polyisocyanate was based on toluene diisocyanate (2,4-TDI) and the polyol was an aromatic with an ester function. These components were furnished by Rexor Company (38, France).

The mixture of isocyanate and polyol, with a stoichiometry 1:1 or 11:100 in mass, respectively, was spread on a silicone sheet of ~25 mm^2^ area and a thickness close to 100 μm. The reaction between the two components was carried out at 70°C under FTIR spectroscopy control. At this temperature, ~80 h were necessary to achieve a conversion of 90% of the isocyanate function. After crosslinking, the adhesive system was analyzed by dynamic mechanical analysis. Two main characteristics were measured. The principal relaxation *T*_α_ at 1 Hz, associated with the glass transition temperature, was determined at (20 ± 5)°C. The storage modulus *E*′ at 70°C (aging temperature) was determined at 10 MPa.

##### Barrier laminate

**Figure 1:**
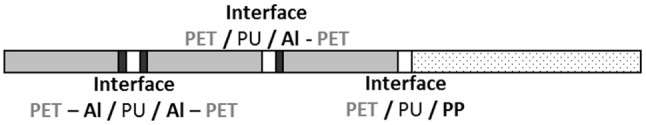
Scheme of multilayer structure with interfaces identification (gray = PET; white = adhesive; black = aluminum; dots = PP).

The barrier envelopes used in this study were provided by Rexor Company (38, France). They were composed of three polyethylene terephthalate (PET) layers of 12 μm thickness coated with 80 nm of aluminum (Al) by physical vapor deposition. The metal was heated and evaporated under vacuum. This condensed on the cold polymer film running on two drums (spools) that was unwound near the metal vapor source. An additional polypropylene (PP) layer of 50 μm thickness was added for weldability. The various films were assembled with 2 μm adhesive layers. The adhesive was deposited by a heliogravure process and the assembly of the different layers was carried out by heat calendaring. Figure 1 shows the studied laminate structure.

The production started with the lamination of two metallized PET layers, with the aluminum layers face to face. Then, the third metallized PET layer and the sealing layer were rolled successively. At each step, fresh glue was exposed a few seconds at 120°C to ensure evaporation of the solvents and water and to initiate the crosslinking. The multilayer was eventually stored at ambient temperature for 5 days to complete the PU crosslinking.

#### Aging

To understand the effect of water and temperature on the interface properties, accelerated aging was performed in a climatic chamber regulated at 70°C and at 90% RH on the laminate and the self-supporting adhesive. In the case of the laminate, rectangular samples (A4 format) were aged.

Some samples of the laminate and the self-supporting adhesive were extracted from this environment after various aging times up to 200–300 or 900 days depending on the nature of samples (laminate or adhesive). Then, samples were taken at the center of each extracted sample to overcome the edge effects that occur during aging, and were characterized by scanning electron microscopy (SEM), Raman microscopy, and FTIR-ATR analyses.

### Methods

#### Scanning electron microscopy (SEM)

SEM was used to observe the different interfaces after aging.

The samples taken on the laminate for different aging times were each placed in a hollow cone that was further filled with epoxy resin. Resin curing was carried out at ambient temperature for 12 h. Then, the cone surfaces were cut with a microtome to reveal the different layers. Microscopy exams were performed on a LEO STEREOSCAN 440 SEM of the ASTRE platform (Savoie Mont Blanc University). A very thin layer of gold was applied onto the samples to prevent charging in the SEM. The cross-sections of the multilayer were observed in the images of back-scattered electrons at 15 kV accelerating voltage and with a magnification × 2000.

#### Spectroscopy

Fourier-transform infrared (FTIR) spectroscopy and Raman microscopy allowed the analysis of chemical functions present in the material. Consequently, it was possible to identify each layer in the laminate and to track the changes in the chemical structure after aging.

##### Infrared spectroscopy in reflexion mode

FTIR-ATR measurements were performed with a Perkin Elmer spectrometer, Paragon 1000, equipped with a single reflexion device (PIKE Diamond MIRacle, Pike Technologies, Madison). All spectra were obtained in the 600–4,000 cm^−1^ frequency range with 16 accumulations and using a spectral resolution of 4 cm^−1^. This technique allows a direct analysis of the surface sample, without requiring any specific preparation.

The penetration depth *d*_*p*_ of the infrared radiation, in the ATR mode, depends directly on wavelength λ, the characteristics of the device (θ, the angle of internal reflection; *n*_*c*_, the refractive index of crystal), and on the physical properties of the sample (*n*_*s*_, the refractive index of sample) according to the following equation (Equation 1).

(1) $$\begin{array}{l} d_{p}=\frac{\lambda }{2\pi n_{c}{\left(\sin^{2}\theta -{\left(\frac{n_{s}}{n_{c}}\right)}^{2}\right)}^{1/2}} \end{array}$$

(Chakrabarti et al., 1999)

As a result, the infrared radiation accesses only the sample surface with a penetration depth up to 2.0 μm.

##### Raman microscopy

As for SEM analyses, the multilayer were embedded in epoxy resin and mechanically polished to reveal the different layers.

Prepolishing was first performed to obtain a flat surface with a minimum of damage. For this, a support with a fixed and relatively coarse particle size was used (foil coated with silicon carbide grains). Polishing was then performed on the flexible fabric support with a diamond-type abrasive whose grains are increasingly fine (up to 1 micron), to obtain a surface without scratches and without deformation. All these steps were performed on an automatic polisher Struers® at low speeds (<150 rpm).

The micro-Raman measurements were performed on a confocal Raman microscope Senterra (Bruker) equipped with an excitation laser at 532 nm that delivered 20 mW to the sample. Light was guided through a dry objective (Olympus, × 100, NA = 0.90), enabling a spatial resolution close to 1 μm and a depth resolution of 2.7 μm. The scattered light was focused through a pinhole (50 μm width) and dispersed onto a DU 420A-DE Andor CCD detector. All spectra were collected in the 3,200–600 cm^−1^ frequency range, with 20 accumulation times of 5 s. A computer controlled the xy stage, allowing increment steps of 1 μm for the profile. Spectra were analyzed with OPUS software.

## Results

### Analysis of degraded self-supporting PU adhesive samples

During aging at 70°C and 90% RH, the appearance of the PU adhesive sample changed gradually, up to 300 days with a viscosity reduction. The sample color varied from bright yellow, originally, to a brownish hue. This alteration has been reported in the literature to be the result of thermal aging of the PU because of the aromatic isocyanates (Salazar and Pack, 2002; Salazar et al., 2003).

#### Molecular modifications

IR spectra were used to track the chemical changes during aging. The possible degradation mechanisms behind these chemical changes are discussed hereafter.

**Figure 2:**
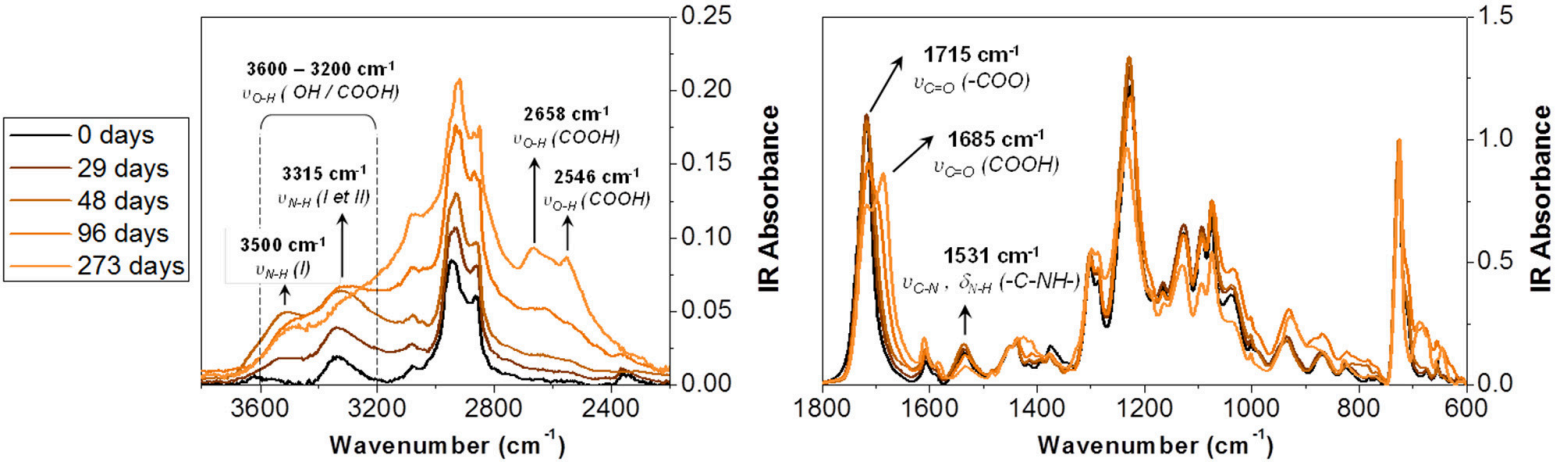
IR spectra of PU adhesive for different aging times.

Figure 2 presents the IR spectra for the self-supporting PU adhesive used as the reference. The spectra were normalized to the 720 cm^−1^ band assigned to a CH_2_ rocking link in the polyol.

In the spectral range between 3,600 and 2,400 cm^−1^, absorption gradually increases during accelerated degradation. This suggests the formation of alcohol and acid groups. The development of two peak shoulders close to 2,550 and 2,660 cm^−1^ were ascribed to the hydroxyl bond (υ_OH_) of the carboxylic acid function. Besides, two bands emerged at 3,315 and 3,500 cm^−1^ with aging. The first one was already observable in the initial spectrum. It corresponds to the N-H vibration of primary amide groups (named NH(II) in Figure 2). The second band was formed during aging and corresponds to the N-H in the primary amine groups (named NH(I) in Figure 2). The formation of this bond also results in an increase of the absorbance toward 3,315 cm^−1^. The degradation of the PU was also revealed between 1,800 and 1,600 cm^−1^, in the spectral region of the carbonyl stretching vibration (υ_C = O_). After 273 days of aging, two bands were visible. The first one, at 1,715 cm^−1^, corresponds to carbonyl related to the ester function of urethane or polyol groups. The second absorption band at 1,685 cm^−1^ can be associated to carbonyl linked to carboxylic acids (COOH). Indeed, compared to the literature (Gulmine et al., 2003; Du et al., 2014; Dubelley et al., 2017b), the carbonyl stretching vibration of carboxylic acids appears 20 cm^−1^ lower compared to the associated ester. The formation of carboxylic acids during aging was confirmed by the gradual increase of the absorption band at 1,408 (δ_OH_), 1,280 (υ_OH_), and 930 cm^−1^ (γ_OH_).

**Scheme 2:**
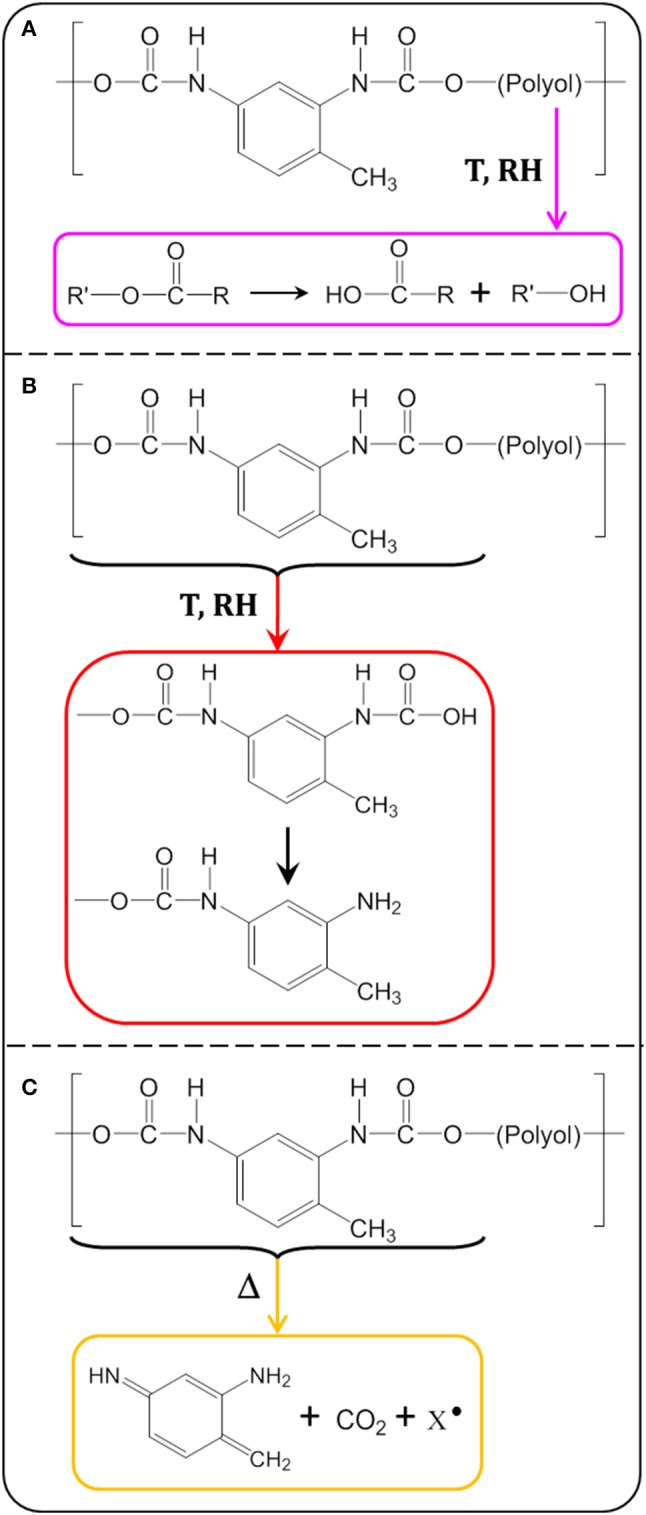
Possible chemical reactions involved in PU adhesive degradation: **(A)** polyol hydrolysis, **(B)** urethane hydrolysis, and **(C)** urethane photolysis.

The formation of carboxylic acid confirms the hydrolysis of the PU adhesive at 70°C and 90% RH. The ester group of the soft segment (polyol) and the urethane group of the hard segment are both susceptible to hydrolysis over time. The ester group was hydrolyzed to reform the acid and alcohol precursors of the polyol (Pellizzi et al., 2014; Scheme 2A). The urethane group can be hydrolyzed to form carbamic acid and precursor alcohol. The carbamic acid formed is unstable and transforms to the primary amine formation with the release of CO_2_ (Scheme 2B; Cauich-Rodríguez et al., 2013). This reaction could be confirmed by the increase of the absorption band at 3,500 cm^−1^, assigned to the N-H stretching vibration of the primary amine groups (Figure 2).

A decrease was also evidenced for the band at 1,531 cm^−1^. It had been assigned to the coupling of the N-H bending vibration with the C-N stretching vibration in the -C-NH group (amide band; Rosu et al., 2009). It was reported to be altered by the photo-oxidation of the PU bases of aromatic isocyanates. The urethane changed its structure and took a stronger chromophore conformation. Rosu et al. (Rosu et al., 2009) showed that the signal area from 1,531 cm^−1^ was linearly correlated with ΔEa,b^*^ (the color difference of yellowing measured by CIELab analysis). The lower the intensity, the greater the color difference. The oxidation of the urethane groups can lead to a strong chromophore structure responsible for the yellowing of PU. From the photo-oxidation mechanism, a possible mechanism of TDI-based PUs thermal degradation was proposed in Scheme 2C (Hoyle and Kim, 1986). The degradation proceeds as a two-step process, giving first the primary aromatic amine products followed by a subsequent reaction to give a quinoid-type compound.

#### Degradation kinetic

Due to the PU adhesive coloring for the advanced states of degradation, only FTIR spectroscopy was used to monitor the degradation. Raman spectroscopy could also be used, but only for the characterization of short aging times. For large degradation, the orange coloration induced a large fluorescence emission, preventing a good acquisition.

The bands of the carbonyl group at 1,685 and 1,715 cm^−1^ were chosen to follow the ester hydrolysis of the polyol or urethane group. The band at 3,500 and 1,531 cm^−1^ allowed to monitor, respectively the urethane hydrolysis and the formation of the colored product by thermal urethane degradation.

**Figure 3:**
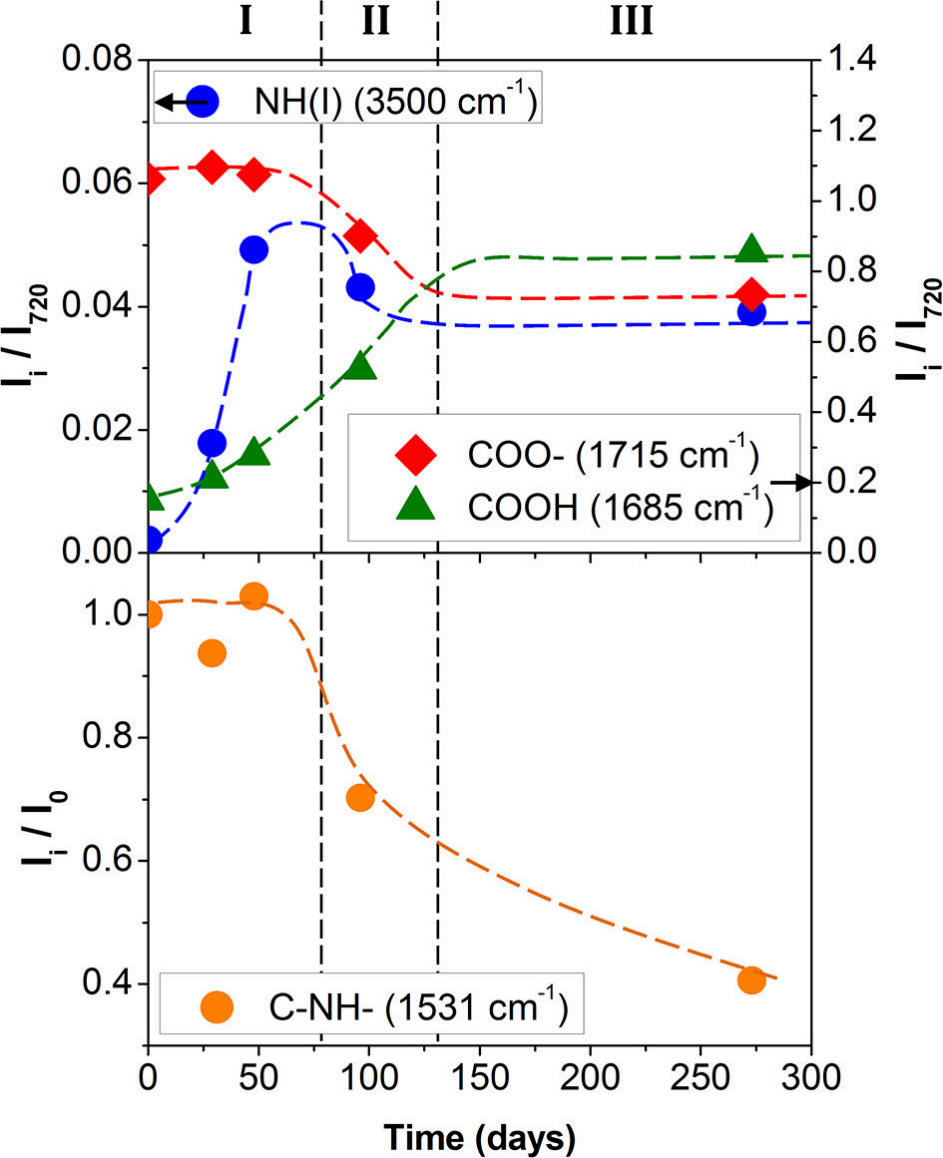
Polyurethane kinetic degradation top) of hydrolysis (• blue: NH(I) 3,500 cm^−1^; green: COOH 1,685 cm^−1^; red: COO– 1,715 cm^−1^) and bottom) of photolysis (• orange: C–NH– 1,531 cm^−1^; Dotted lines are guidelines).

In Figure 3, the normalized intensity of the absorption bands, linked to hydrolysis, are plotted as a function of the accelerated degradation time. The development of the absorption band at 1,686 cm^−1^ as well as the decrease of the absorption intensity at 1,715 cm^−1^ clearly reflected the hydrolysis of the ester function of the polyol or urethane group. In parallel, the increase of the absorption intensity at 3,500 cm^−1^ indicated urethane hydrolysis. The three hydrolysis markers appeared to stabilize after 150/200 days of aging at 70°C/90% RH. This indicates a very large degradation of the PU adhesive.

Although former studies agree that the urethane function is less sensitive to the ester hydrolysis of polyol (Gac et al., 2013; Pellizzi et al., 2014), it seems that two hydrolysis mechanisms are concomitant. The band at 3,500 cm^−1^ is unfortunately located in a region also affected by the vibrations of the hydroxyl bonds linked simultaneously to carboxylic and carbamic acids, alcohols, and residual water. The kinetics and the chronology for the two hydrolysis mechanisms remain hard to estimate.

Figure 3 shows the ratio *I*_*i*_*/I*_0_ of the signal intensity from 1,531 cm^−1^ (amide II band), where I_i_ is the band intensity measured for the PU degraded sample and *I*_0_ the band intensity for the nondegraded sample. The systematic decrease of the I_i_/I_0_ ratio was in agreement with the Rosu et al. (Rosu et al., 2009) analyses and supports the idea that the urethane linkage undergoes rearrangement.

The macroscopic observations and IR analyses revealed a marked deterioration of the self-supporting PU adhesive, even for durations lower than 50 days. Several mechanisms were involved, leading to three stages as a function of time. This chemical degradation was accompanied by a loss of viscosity that indicated a significant modification of the mechanical properties and a reduction of the network density. Indeed, Boubraki et al. highlighted a reduction of ~30% in tensile stress and modulus after aging at 70°C on immersion in water for 6 months. A reduction of 44% in shear modulus was also observed after 12 months of immersion in water at 40°C (Sousa et al., 2018). However, in our study, these macroscopic observations could not be measured due to the brittleness of the aged samples.

### PU degradation in multilayers

#### Delaminations

**Figure 4:**
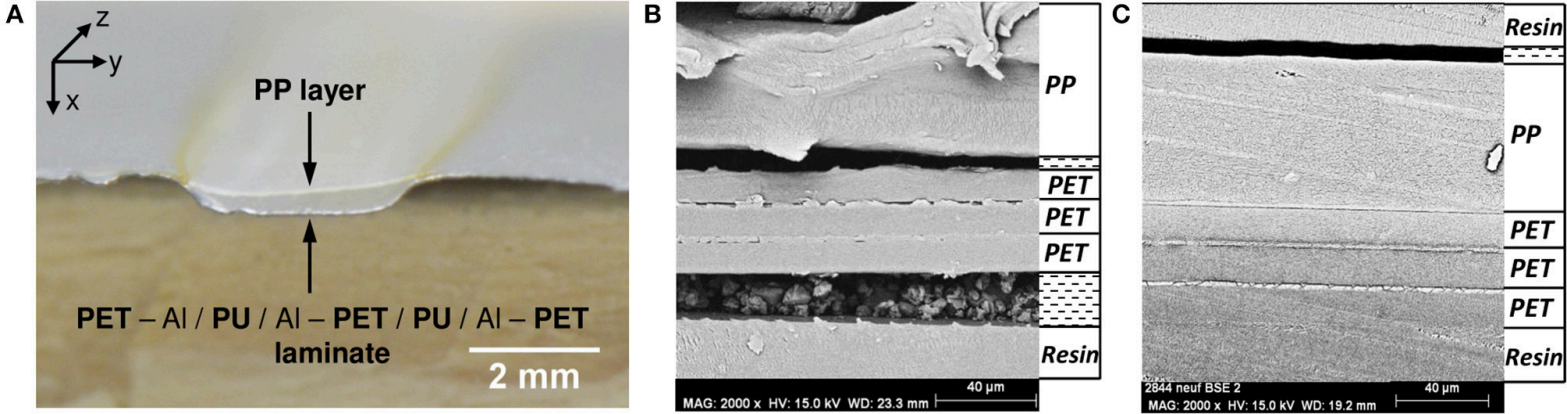
**(A)** Picture of the edge of the laminate after 192 days at 70°C/90% RH illustrates the delamination, **(B)** micrograph of the cross section and identification of each layer in delaminate area, and **(C)** micrograph of the cross section of laminate before aging.

Multilayers (Figure 1) were aged at 70°C and 90% RH during 192 days. After 192 days, small and localized delaminations could be observed on the entire rectangular sample. Figure 4A shows a macroscopic observation of delamination formed at the center of the sample. A cut was performed in this sample to probe the layer integrity to better localize decohesions. SEM analyses were performed (Figure 4B). The different polymer layers could be clearly identified. Figure 4B shows that the three PET layers remained glued to one another and that the delamination occurred at the PP/PU/PET interface. By comparison with Figure 4C, one can safely conclude that this delamination was produced by thermal aging and not sample preparation. Indeed, Figure 4C shows the cross-section of the multilayer before aging and the sample was prepared in the same conditions. The three PET layers and the sealant remained glued to one another. The delamination in this polyolefin–polyester interface is strongly related to the shrinkage of the PP film. Indeed, a heat treatment of this film at 70°C during 192 days can induce a noticeable shrinkage of approximately −0.7% (Dubelley et al., 2017a). These authors showed that for such a shrinkage level, the PET/PU/PP interface was delaminated. More specifically, the difference of the shrinkage between two films seems to control the appearance of defects. The strength of the adhesive is, however, likely to play an important role as well. To differentiate between adhesive and cohesive rupture, the PP/PU/PET interface was analyzed with Raman microscopy.

**Figure 5:**
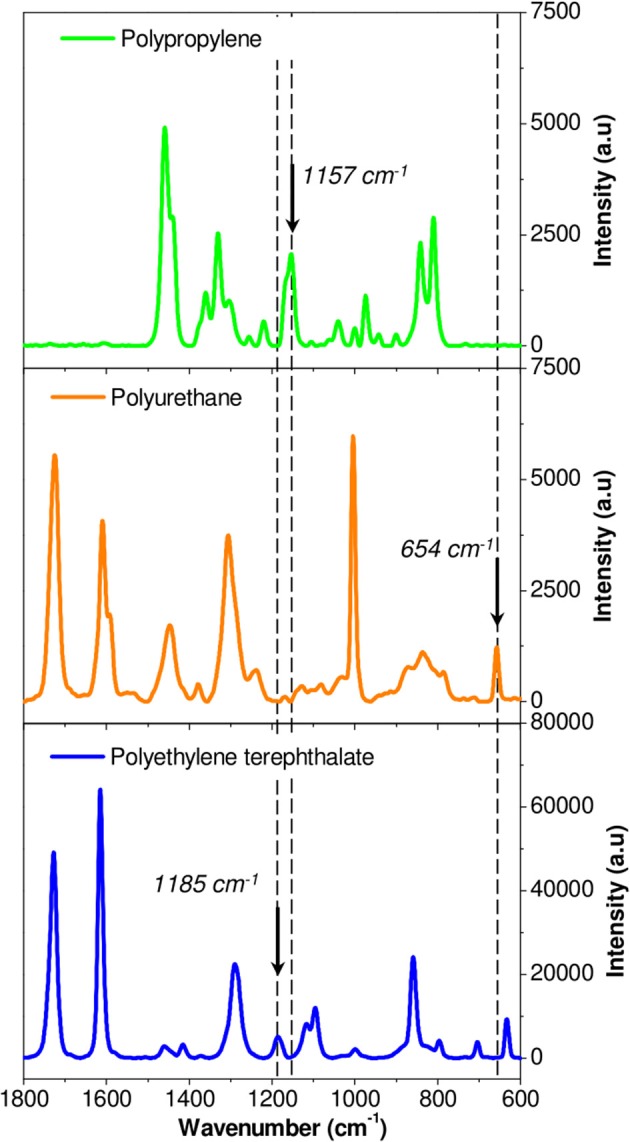
Identification of specific band for each polymer in Raman spectroscopy: Polypropylene (green line), Polyurethane (orange line), and Polyethylene terephthalate (blue line).

**Figure 6:**
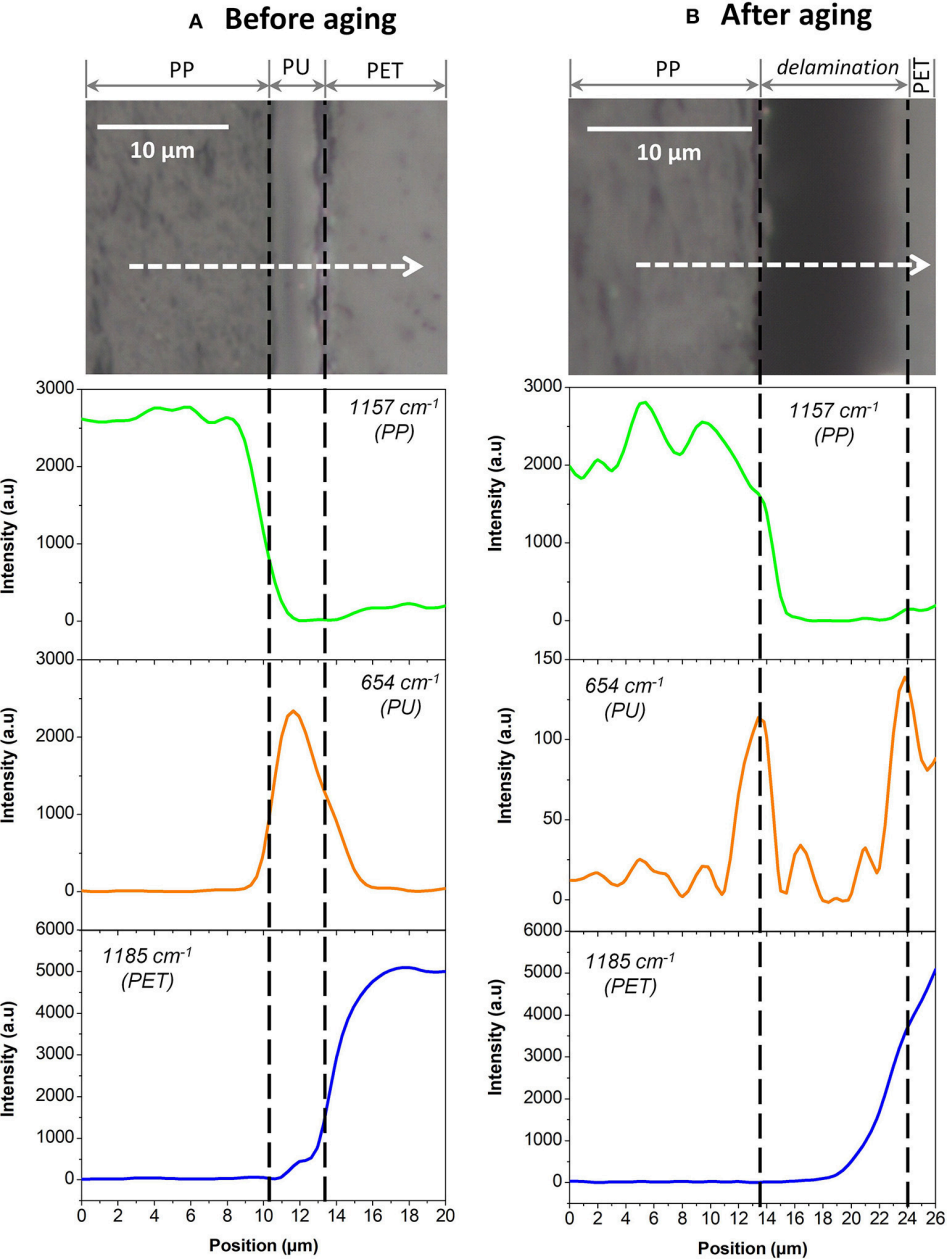
PP/PU/PET interfaces analyzed by Raman microscopy **(A)** before aging and **(B)** after aging. At the top, micrographic observations of interfaces. At the bottom, the results of the three components 654, 1,157, and 1,185 cm^−1^, respectively in green, orange, and blue.

Each component was first studied independently. Figure 5 shows the Raman spectra of unaged PP, PU, and PET between 1,800 and 600 cm^−1^. Unfortunately, the spectral responses of the three polymers largely overlap in this region. Specific bands could however be identified: 654 cm^−1^ for the PU, 1,185 cm^−1^ for PET, and 1,157 cm^−1^ for PP. The PP/PU/PET interfaces, before and after aging at 70°C/90% RH during 192 days, were then characterized along a line through the interface every μm (Figure 6). A detailed analysis of the spectra was performed with the three components identified previously (PU, PP, and PET). Figure 6 shows the intensity of the three bands (654, 1,157, and 1,185 cm^−1^) along the profile before and after aging. The three layers could be easily identified before aging. The continuity of the adhesive layer was observed with the image and confirmed with the chemical compositions. After aging, a gap of 10 μm width was observed on the micrograph, corresponding to a delamination. By comparison with the micrograph before aging, we could safely conclude that this delamination was produced by thermal aging and not sample preparation. More interestingly, a band at 654 cm^−1^ confirmed the presence of the adhesive on both sides. The delamination at the PP/PU/PET interface resulted from the cohesion failure of the adhesive PU. The yellowing of the adhesive at the edge of the delamination (Figure 4A) suggests a chemical degradation during the accelerated aging of the complex.

#### Chemical analyses

##### Polymer–polymer interfaces (PET/PU/PP)

**Figure 7:**
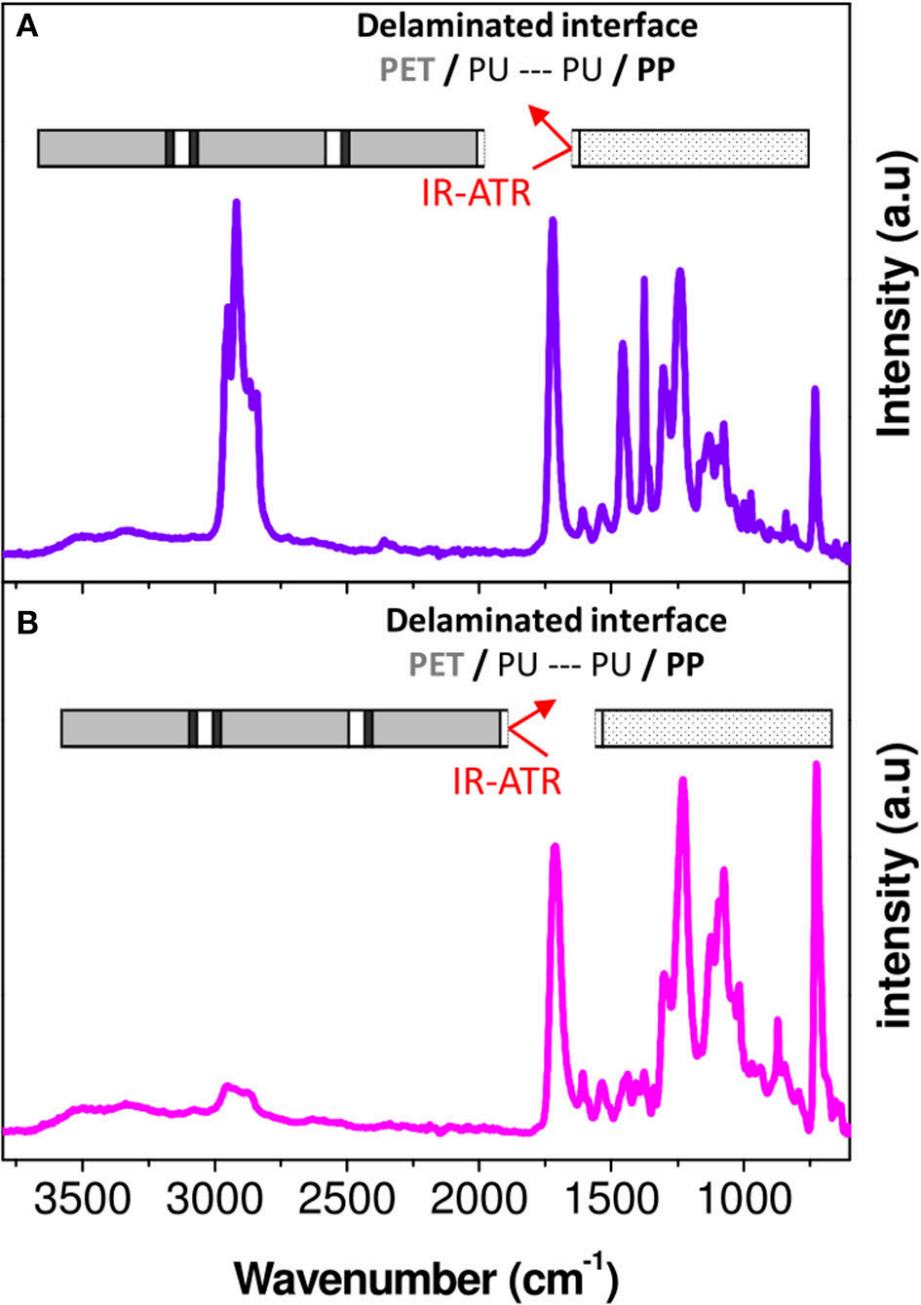
FTIR-ATR spectra corresponding to PP **(A)** and PET **(B)** surfaces obtained after manual peeling of interfaces PP/PU/PET.

For the purpose of revealing the chemical changes of the PU adhesive in the multilayer after aging, the latter must be characterized locally. For this, the layers of PET and PP were separated manually in the delamination areas. Then, the adhesive parts on the PP surface and on the PET surface were analyzed by FTIR-ATR (Figure 7).

**Figure 8:**
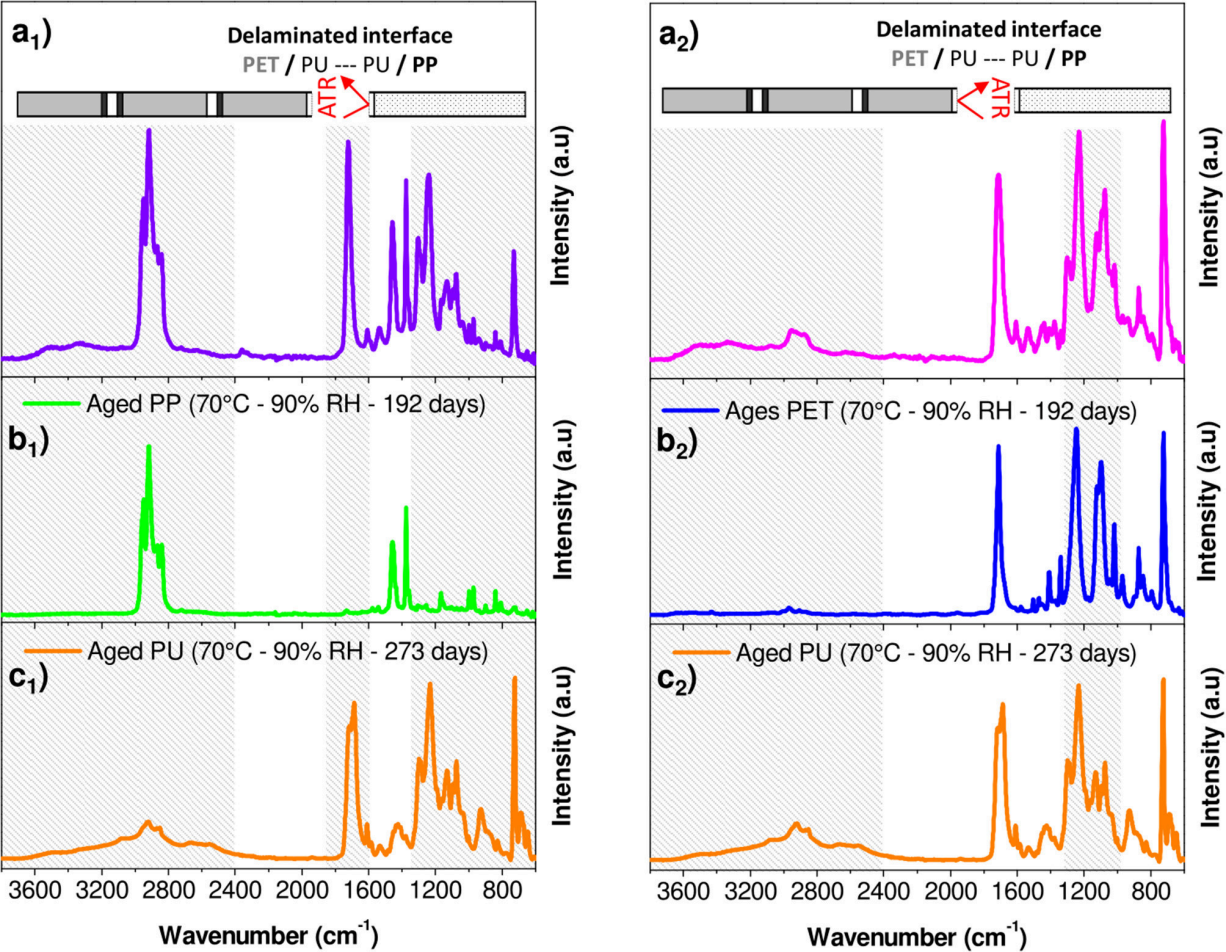
FTIR-ATR spectra corresponding to **(a**_1_**)** PP surface obtained after manual peeling of interfaces PP/PU/PET; **(a**_2_**)** PET surface obtained after manual peeling of interfaces PP/PU/PET; **(b**_1_**)** Aged PP at 70°C/90% RH during 192 days; **(b**_2_**)** Aged PET at 70°C/90% RH during 192 days; **(c**_1_**,C**_2_**)** Aged PU at 70°C/90% RH during 273 days.

A rapid screening of the IR spectrum obtained for the PP surface in comparison with the IR spectra of the aged PP and PU revealed the presence of the bands between 1,400 and 600 cm^−1^ and between 1,800 and 1,600 cm^−1^ (Figures 8a_1_,b_1_,c_1_). An additional broad band with a high intensity could be also observed in the 3,800–2,400 cm^−1^ region. This confirmed the presence of the adhesive on the PP surface. In contrast, the PET surface appeared more complex, essentially because the spectral responses of PET and PU overlap (Figures 8a_2_,b_2_,c_2_). However, a broad band in the 3,800–2,400 cm^−1^ region and a difference in the band shape in the 1,400–1,000 cm^−1^ region can be the evidence of the adhesive's presence on the PET surface.

To determine the amount of the adhesive on both the substrates (PP and PET surfaces) and the progress of its degradation, a mathematical decomposition of the signal had thus to be used. Because of the cohesion failure mechanism appended on very thin layers (2 μm) both the film support (PET or PP) and adhesive PU should be reached by the IR beam. The spectrum of each delaminated surface was assumed to be the weighted sum of the spectra of each constituent, the support (PET or PP) and the adhesive. In addition, the aging of each component must be considered.

The spectral responses at the PET and PP surfaces were decomposed into four components, fresh and aged supports and fresh and aged adhesives (Equation 2):

(2) $$\begin{array}{l} {A\left(\sigma \right)}_{interface}=\sum {A\left(\sigma \right)}_{x}{\;\Phi }_{x}+\sum {A\left(\sigma \right)}_{xa}{\;\Phi }_{xa} \end{array}$$

where *A(*σ*)*_*interface*_ is the experimental absorbance of a given surface for each wavenumber σ, *A(*σ*)*_*x*_ is the absorbance of fresh components, and *A(*σ*)*_*xa*_ is the absorbance of aged components. The weight fraction ϕ_*x*_ and ϕ_*xa*_ are respectively for fresh and aged constituents (PET or PP and PU). They are the only fit parameters and were determined by minimizing the residual sum of squares between the experimental data and the model. As a result, the individual ϕ_*i*_ may be viewed as an individual amount of each component.

**Figure 9:**
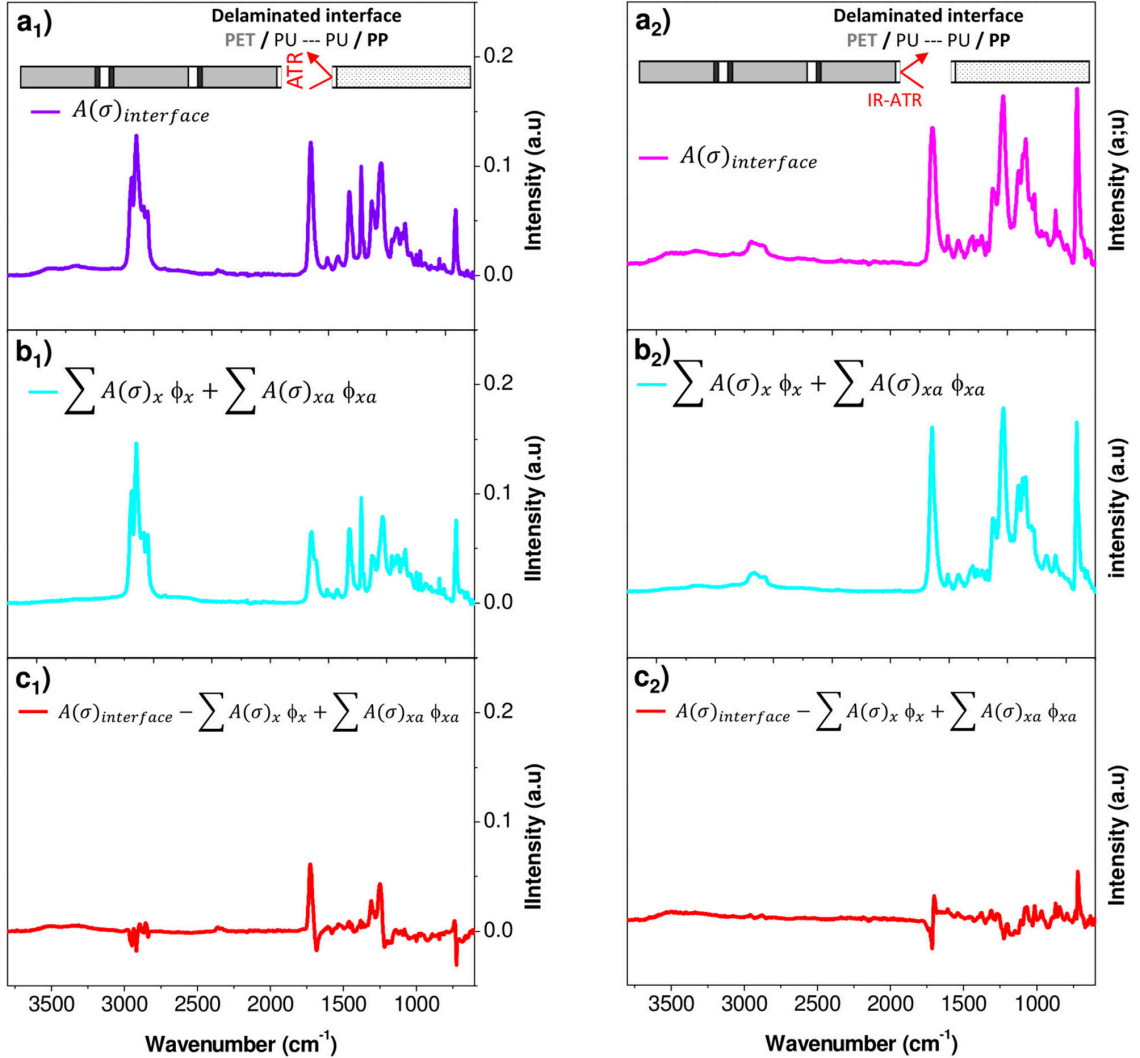
Factorization of IR spectral responses in PP (_1_) and PET (_2_) surfaces after delamination **(a)** PP or PET surface analyzed, **(b)** PP or PET surface calculated with Equation (2), and **(c)** residue.

**Figure 10:**
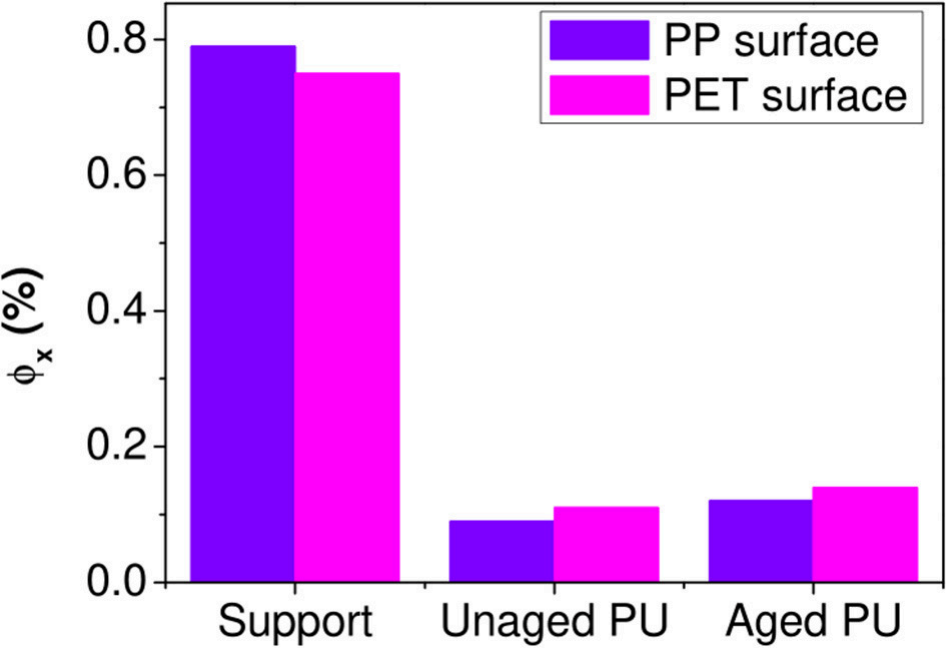
Weight fractions ϕ_x_ of constituents in analyzed PP and PET surfaces.

An example of spectrum decomposition is given in Figure 9 for the PP surface. Figure 9 shows the experimental IR spectrum of the PP surface characterized (Figure 9a_1_), the calculated IR spectrum of the surface obtained with the resolution of Equation (2) (Figure 9b_1_) and the residue (Figure 9c_1_) obtained by subtracting the two previous spectra. The residue took essentially the form of a noisy signal, without a significant band revealing that the model contains all the needed inputs. The same decomposition was conducted on the PET surface (Figures 9a_2_,b_2_,c_2_). Figure 10 shows the weight fraction of the constituents determined on the PET and PP surfaces. The IR spectra of PET and PP did not show a significant difference with aged samples. It means that the amount of aged PP and PET should therefore be fixed as 0. This assumption allows to reduce the number of independent variables and stabilizes the calculations. For both supports, fresh and aged PU represented 10 and 13%, respectively of the spectra.

The results first confirmed the presence of PU on either side of the fissure and thereby the cohesive rupture of the thin glue layer in the polyolefin–PET interface. To determine whether PU has or not undergone a chemical degradation, the relative amount of the aged component was used. In both decompositions, ϕ_*PUa*_ represents ~60% of the total weight fraction of PU. An estimation of the equivalent aging time of the PU layer encapsulated at the interface can be realized using the calibration curves obtained on self-supporting films (Figure 3). The adhesive degradation in the multilayer structure after 192 days of aging at 70°C and 90% RH reached state II. Delaminations could also be observed in the metallic bonded interfaces, PET-Al/PU/Al-PET and PET-Al/PU/PET (Figure 1) and this after a longer degradation time.

##### Polymer–metal interfaces

**Figure 11:**
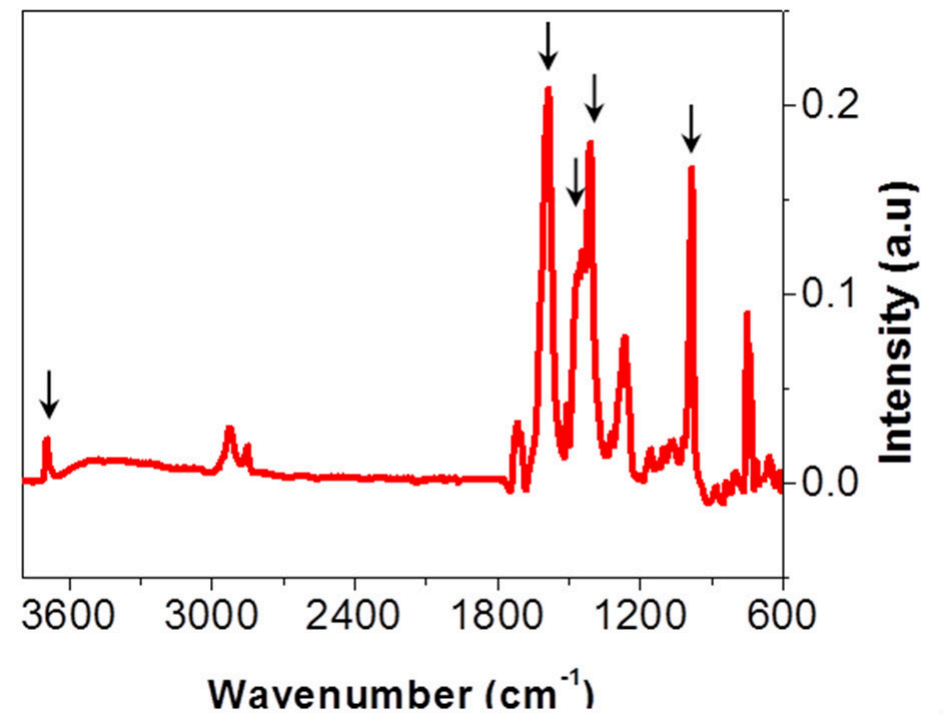
Residue extracted from PET-Al/PU/PET interfaces analyses obtained using Equation (2).

**Scheme 3:**
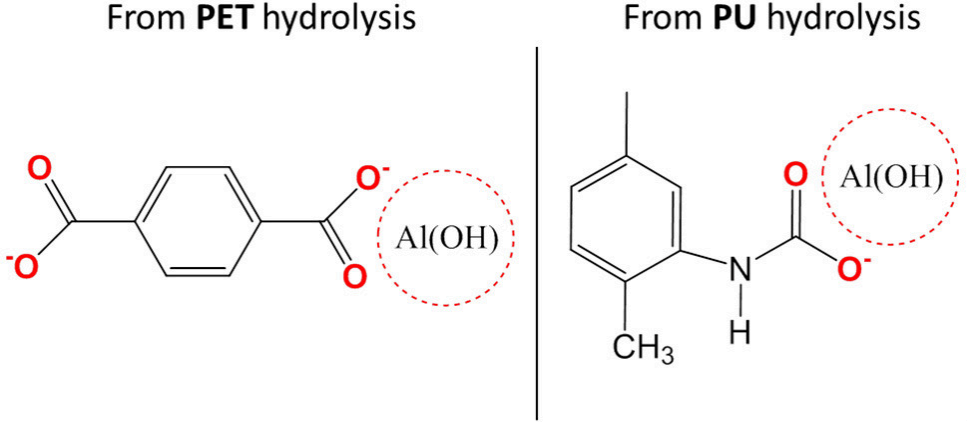
Schema of aluminum terephthalate ([Al(OH)(1,4-benzenedicarboxylate)]_n_) and aluminum carbamate.

Studied multilayers (Figure 1) present delaminations in the PET-Al/PU/PET and PET-Al/PU/Al-PET interfaces after 870 days of aging at 70°C and 90% RH. In both cases, delaminations did not concern the interface between PET and the deposited aluminum. They were identified as cohesive by the same methods presented so far and the PU adhesive at the interface had reached state III of degradation. Nevertheless, Equation (2) does not fit the experimental data. The signal decomposition of polymer or aluminum surfaces after delamination led to a positive residue. This residue is shown in Figure 11 and can be compared with the residues obtained after signal decomposition of the delaminated PET/PU/PP interface (Figures 9c_1_,c_2_). It seems that there was a degradation product on the PET and aluminum surfaces. The residue exhibited a thin peak at 3,700 cm^−1^ that might be ascribed to the vibrational band of the free hydroxyl group ν_O−H_ combined with metal (Rallapalli et al., 2011; Zhang et al., 2012). The hydroxyl group vibration was also visible at 980 cm^−1^. Strong vibrational bands were noticeable in the region of 1,700–1,400 cm^−1^ that were assigned to the carboxyl function. The band at 1,580 cm^−1^ corresponded to –COO- asymmetric stretching whereas the bands at 1,410 and 1,450 cm^−1^ were attributed to –COO- symmetric stretching. According to the literature (Meilikhov et al., 2010), this residue could be matched with aluminum terephthalate ([Al(OH)(1,4-benzenedicarboxylate)]_n_; Scheme 3).

The product of degradation could result from the dissolution of the aluminum layer. This dissolution can be explained by the hydrolysis of PET or the PU adhesive. PET hydrolysis is a well-known phenomenon (Dubelley et al., 2017b). The authors reported the hydrolysis of PET in the multilayer in the same condition that leads to the formation of carboxyl group by chain scission after 400 days of aging. These scissions can lead in the long term to terephthalic acid formation. The results from the current work showed that the PU can be hydrolyzed given a carbamic acid throughout the intermediate step (Scheme 2B). Both products—terephthalic acid and carbamic acid—can be responsible for a pH decrease in the metallized interface. In this confined space, the pH change may contribute to the depassivation of the latter with Al^3+^ formation (Davis, 1999). Ions can then combine with the acids and form aluminum terephthalate or aluminum carbamate (Scheme 3).

**Figure 12:**
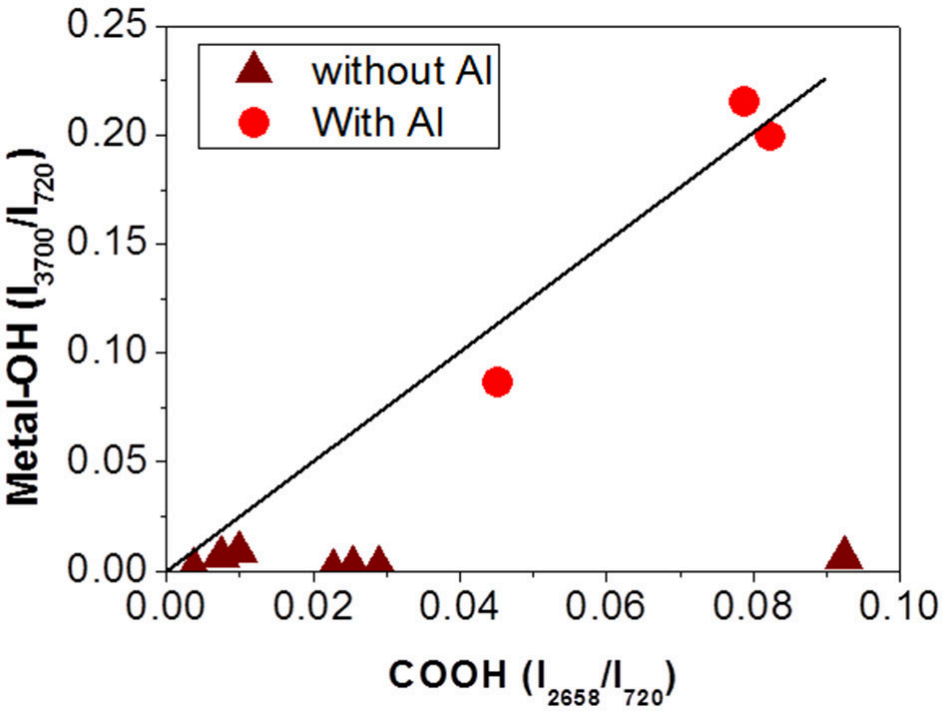
Characterization of metallized (• red) or not metallized (wine) interfaces by IR spectroscopy. Evolution of IR intensity of metal-OH bond with IR intensity of acid carboxylic (solid line is a guideline).

Analyses of the different interfaces, metallic or not, for various aging times were conducted. Figure 12 summarizes the results of these analyses. Figure 12 presents the evolution of the IR intensity of the free hydroxyl group I_3700_/I_720_, metal-OH bond marker, with the evolution of the IR intensity of carboxylic acid (COOH) I_2658_/I_720_, PET and PU degradation's marker. The degradation product combined with Al was formed in the presence of acid and only in metallic interfaces. In addition, the concentration of the degradation product increases with the state of degradation.

## Conclusion

First, the hygrothermal degradation of auto-supported PU adhesive at 70°C and 90% RH was studied during ~9 months. The macroscopic FTIR-ATR study of PU allowed highlighting the existence of several markers characteristic of the PU hydrolysis. The most relevant markers were (i) the coupling 1,686 and 1,715 cm^−1^ bands for the carboxylic acid formation as a consequence of a major phenomenon of chain scission, and (ii) the 1,531 cm^−1^ band for the coloration of samples, characteristic of the loss of the PU structure. The PU hydrolysis first occurred very quickly and then leveled off. It seemed complete after 150/200 days of hygrothermal aging.

Secondly, the analyses of the multilayer interfaces were performed using Raman spectroscopy and FTIR-ATR spectroscopy. These two techniques permitted to identify the interface delamination as a cohesion failure of the adhesive. A more detailed analysis made it possible to demonstrate that the PU adhesive was degraded even if the PP and PET films do not undergo significant chemical degradation. The stage of degradation depends on not only the aging time but also the type of interface (polymer–polymer or metal–polymer). In case of the polymer–metal interface, degradation's product results from the oxidation of the aluminum layer by PU or PET hydrolyses have also been highlighted. The adhesive between the films would play an important role in the failure of the multilayer stack. It appears as one of the limiting laminate components coupled to films shrinkage. Indeed, the intrinsic properties of the adhesive and its behavior during aging may affect not only the adhesion properties but also the mechanical strength of the PU.

From an application point of view, the various delaminations as well as the aluminum depassivation will lead to a loss of laminate barrier properties. This will lead to a water build-up by the VIP silica core. In the long term, the conductivity of the VIPs will increase and the VIP will be no longer considered as a super-insulating system.

## Author contributions

FD: experimental tasks and interpretations. CB: FTIR interpretation. EPl: Raman interpretation. EPo: aging sample preparation. BY: industrial supervisor. LF: academic supervisor.

### Conflict of interest statement

The authors declare that the research was conducted in the absence of any commercial or financial relationships that could be construed as a potential conflict of interest.
